# Effect of Intra-Canal Calcium Hydroxide Remnants on the Push-Out Bond Strength of Two Endodontic Sealers

**DOI:** 10.22037/iej.2017.33

**Published:** 2017

**Authors:** Sholeh Ghabraei, Behnam Bolhari, Fatemeh Yaghoobnejad, Naghmeh Meraji

**Affiliations:** a *Department of Endodontics, Dental School, Tehran University of Medical Sciences, Tehran, Iran; *; b *Private Practice, Tehran, Iran*

**Keywords:** AH-26, Calcium Hydroxide, Endosequence BC Sealer, Push-Out Bond Strength

## Abstract

**Introduction:**

The aim of this study was to evaluate the effect of intra-canal calcium hydroxide (CH) remnants after ultrasonic irrigation and hand file removal on the push out bond strength of AH-26 and EndoSequence Bioceramic sealer (BC Sealer).

**Methods and Materials:**

A total of 102 single-rooted extracted human teeth were used in this study. After root canal preparation up to 35/0.04 Mtwo rotary file, all the specimens received CH dressing except for 34 specimens in the control group. After 1 week, the specimens with CH were divided into 2 groups (*n*=34) based on the CH removal technique; *i.e.* either with ultrasonic or with #35 hand file. Then specimens were divided into two subgroups according to the sealer used for root canal obturation: AH-26 or BC Sealer. After 7 days, 1 mm-thick disks were prepared from the middle portion of the specimens. The push out bond strength and failure mode were evaluated. Data were analyzed by the two-way ANOVA and Tukey’s post hoc tests.

**Results:**

The push out bond strength of both sealers was lower in specimens receiving CH. These values were significantly higher when CH was removed by ultrasonic (*P*<0.05). The dominant mode of failure in all subgroups was of mixed type except for the BC Sealer specimens undergoing CH removal with hand file which dominantly exhibited adhesive mode of failure.

**Conclusion:**

CH remnants had a negative effect on the push out bond strength of AH-26 and BC Sealer. Ultrasonic irrigation was more effective in removing CH.

## Introduction

Calcium hydroxide (CH) is the most widely used intracanal dressing in endodontics due to its antibacterial and biological properties [1]. Complete removal of this intra-canal dressing from the root canal system is very difficult. Several studies have investigated the ability of various techniques (such as different rotary instruments and different irrigating systems and solutions) in its removal [2-4]. For instance, Altunsoy *et al.* [2] found no significant differences between the amount of residual CH in the root canals after the use of ProTaper, Reciproc, OneShape, WaveOne and manual files. None of the instruments were able to completely remove this dressing. Other studies have also concluded that CH was not completely eliminated from the root canal after the application of different irrigating systems and solutions such as passive ultrasonic irrigation, EndoVac, EndoActivator, sodium hypochlorite or ethylenediaminetetraacetic acid (EDTA) [3, 4]. Therefore, encountering residual CH in the root canal system before obturation is inevitable. Residual dressing can affect some properties of endodontic sealers [5-8] and subsequently affect treatment outcomes. 

Endosequence BC Sealer (Brasseler USA, Savannah, GA) is a premixed bioceramic sealer composed of zirconium oxide, calcium silicates, calcium phosphate monobasic, calcium hydroxide, filler, and thickening agents [9, 10]. Its nanoparticle size allows it to flow into canal irregularities and dentinal tubules. It is hydrophilic and uses the moisture in dentinal tubules to initiate and complete its setting reaction [10, 11]. Hedge *et al.* [12] demonstrated that residual CH intracanal medicament subsequent to rinsing with 17% EDTA followed by sodium hypochlorite significantly reduced the push out bond strength of BC Sealer. 

Up to this date no study has evaluated the effect of different CH intracanal medicament removal techniques on the push out bond strength of Endosequence BC Sealer. Therefore, the aim of this study was to evaluate the effect of residual CH after ultrasonic irrigation and hand file removal on the push out bond strength of Endosequence BC Sealer and AH-26 sealer. The null hypothesis was that residual dressing will negatively affect the push out bond strength of these sealers.

## Materials and Methods

One hundred and two maxillary central incisors and canines that were extracted due to periodontal problems and stored in 0.5% chloramine-T were selected. Teeth with immature root apices, root canal curvatures, caries, cracks and resorptive defects in the roots were excluded. The crowns were cut off below the cementoenamel junction to a standardized root length of 15 mm. A #15 K-file was inserted into the root canal until it could be seen at the apical foramen. The working length was determined by reducing this length by 1 mm. The root canals were prepared using Mtwo NiTi rotary instruments (VDW, Munich, Germany) up to a 35/0.04 file. Normal saline was used for irrigation. Thirty four specimens were considered as controls and did not receive CH, whereas, 68 were filled with CH (Golchadent, Karaj, Iran) (which was mixed with normal saline in a 1:1.5 powder to liquid ratio) using a #25 lentulo spiral (MicroMega, Besancon, France) and a low speed handpiece. The coronal portions of all specimens were sealed with sticky wax. After 1 week incubation in 37^º^C and fully saturated conditions, the specimens with CH were divided into 2 groups (*n*=34) according to the method used for CH removal: ultrasonic instrumentation (NSK Varios 350, NSK, Tochigi, Japan) with the power setting at 6 for 30 sec with pull and push movements, or #35 stainless steel hand K-files and 5 mL of normal saline. Afterwards, all root canals were dried by paper points and then divided into 2 subgroups according to the sealer used for obturation: AH-26 (Dentsply, DeTrey, Germany) or BC Sealer (Brasseler USA, Savannah, GA).

In all groups a #40 gutta-percha cone (DiaDent, Chungcheongbuk-do, Korea) was used as the master apical cone and obturation was completed by lateral compaction using a #25 accessory gutta-percha cones. Radiographs of the specimens were taken to confirm the quality of the root canal fillings. Then the excess gutta-percha was removed by a heated instrument and the coronal portion was sealed with temporary filling material (Coltosol, Aria-Dent, Tehran, Iran). The specimens were incubated for 7 days at 37^º^C with 100% humidity.

After mounting specimens in acrylic resin, the middle third of the roots were horizontally sectioned to obtain 1.00±0.05 mm slices using a water-cooled precision saw (Isomet, Buehler Ltd., Lake Bluff, Illinois, USA). Apical and coronal aspects of each slice were then digitally photographed. Afterwards, the circumference of the filling material from the coronal and apical aspects of each slice was calculated using an AutoCAD software program (version 16.0, Autodesk, Inc., San Rafael, CA, USA). The thickness of the root slices were measured using a digital caliper (Digimatic, Mitutoyo Corp., Japan). The interfacial area (in mm^2^) was calculated by the following formula: (coronal circumference + apical circumference)/2 × thickness.

The filling material was then loaded with a 0.7-mm diameter cylindrical stainless steel plunger. The diameter of the selected plunger was smaller than the canal diameter to ensure contact with the cement only. Loading was applied on a universal testing machine (Z050, Zwick/Roell, Ulm, Germany) at a speed of 0.5 mm/min in an apical-coronal direction to avoid any interference due to root canal taper during push out testing. The bond strength was determined using a computer software program connected to the universal testing machine. 

The maximum load applied to the filling material before debonding was recorded in Newtons (N). The load at failure was was divided by the interfacial area to express the bond strength in megapascals (MPa). 

After the bond strength test was performed, both sides of the root slices were examined under a light microscope (Carl Zeiss, fl70, Oberkochen, Germany) at ×25 magnification to determine the failure mode. Modes of bond failure were considered as follows: adhesive; at filling material-dentin interface, cohesive; within filling material, and mixed failure. 

Additional 2 specimens in each main experimental group undergoing CH removal were prepared as mentioned above. After CH removal the specimens were sectioned longitudinally and examined under scanning electron microscopy (SEM) to evaluate the amount of residual CH.

Data were analyzed by two way ANOVA and Tukey’s post hoc test. The level of significance was set at 0.05. 

## Results

The mean±SD of push out bond strength values in different experimental groups are shown in Table 1.

The highest push out bond strength values in both evaluated sealers were seen in the controls (4.41±1.5 and 4.94±1.3 for BC Sealer and AH-26, respectively) while the lowest were seen in the file groups (2.69±1.0 and 3.72±1.8 for BC Sealer and AH-26, respectively). All pairwise comparisons in the BC Sealer specimens showed statistically significant differences (*P*<0.05). When comparing the push out bond strength of AH-26 specimens, no significant difference was seen between the control and ultrasonic groups (*P*=0.9) while the push out bond strength of the control and ultrasonic groups were significantly higher than that of the file group (*P*<0.05).

When comparing the control specimens, no significant difference was seen between different sealer types (*P*=0.8). However, in specimens receiving CH, the push out bond strength of AH-26 was significantly higher than that of BC Sealer regardless of the CH removal technique (*P*<0.05). 

Failure modes in experimental groups are shown in Table 2.

The majority of the specimens obturated with AH-26 exhibited mixed mode of failure. The dominant failure mode seen in the BC Sealer specimens was mixed with an exception of those undergoing CH removal by hand files which exhibited a dominant adhesive mode of failure. The mixed mode of failure was the dominant mode of failure seen in the control groups regardless of the sealer used. 

**Figure 1:**
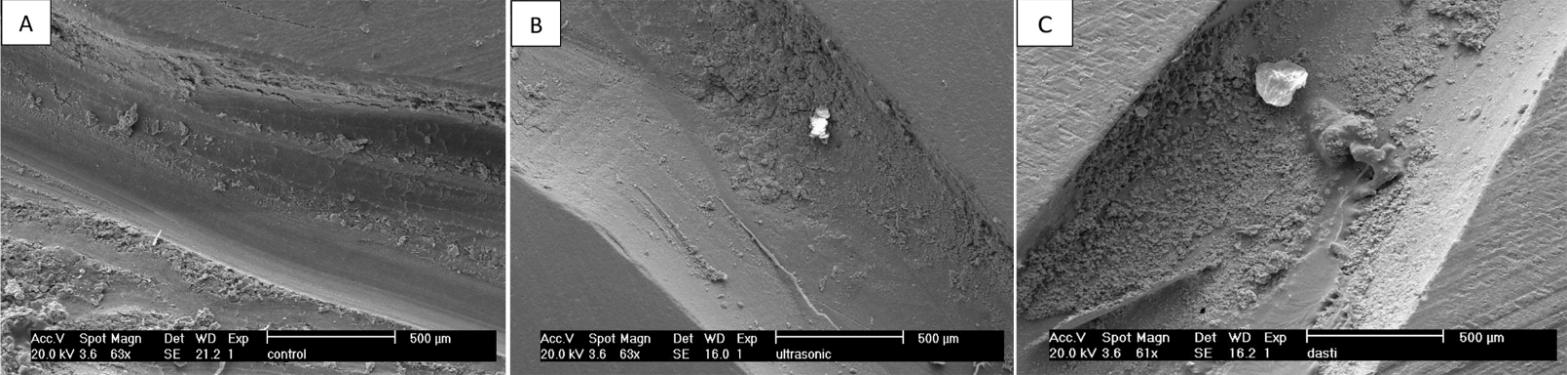
Scanning electron microscopic (SEM) images of residual CH in: *A)* control; *B)* ultrasonic and *C)* file groups

According to the SEM findings (Figure 1), the amount of residual CH in the ultrasonic group was less than that of the hand group.

## Discussion

Adhesion to dentinal walls of the root canal is a basic requirement for root canal sealers [13]. This property is dependent on various factors, including the intermolecular surface energy and cleanliness of dentin, presence of smear layer [14, 15] and the surface tension and wetting ability of the sealer [16]. 

The bond strength of epoxy-based sealers such as AH-26 is attributed to the formation of a covalent bond between an open epoxide ring and exposed amino groups of dentin collagen [17]. Also, the flowing ability of these sealers causes better penetration into root canal irregularities contributing to mechanical interlocking between the sealer and dentin [18]. Furthermore, the slightly acidic pH of these sealers might result in a self-etching effect on dentin resulting in enhanced bond strength [19]. 

The bond between bioceramic sealers such as BC Sealer and dentine has been attributed to the chemical bond developed through the production of hydroxyapatite during setting [20]. Moreover, this hydrophilic sealer has a low contact angle allowing it to spread easily over the dentinal walls leading to adaptation and penetration into root canal irregularities [21]. 

Leaving or removing smear layer in the root canal system is a controversial issue. Some believe that the leaving smear layer may block dentinal tubules and impede bacterial or toxin penetration [22]; while others suggest that it prevents irrigant and obturation material penetration into the dentinal tubules, which increase the risk of infection and microleakage [23, 24]. Studies have shown that smear layer removal did not have a significant effect on the push out bond strength of endodontic sealers [25-27]. Therefore, in the current study smear layer was not removed. 

The push out bond strength of both evaluated sealers decreased in specimens pretreated with CH indicating that residual CH adversely affected the push out bond strength of both sealers. Furthermore, the push out bond strength of both sealers was significantly higher in specimens in which CH removal was performed by ultrasonic irrigation. SEM findings confirmed the presence of less amounts of residual CH in the ultrasonic groups. Neither ultrasonic nor hand files lead to complete CH removal. These findings indicate an inverse relation between amounts of residual CH and push out bond strength values in both sealers. 

Residual CH can act as a barrier, that prevent the development of the chemical bonds between the sealers and dentin and negatively affect their adaptation with dentinal walls; resulting in decrease in the push out bond strength.

**Table 1:** Mean (SD) of push out bond strength values in different experimental groups

	**Mean (SD)**		
	**Control**	**Ultrasonic**	**File **
**AH-26**	4.94 (1.3)	4.65 (1.8)	3.72 (1.8)
**BC Sealer**	4.41(1.5)	3.39 (1.5)	2.69 (1.0)

**Table 2:** Failure modes in experimental groups

	**AH-26**			**BC Sealer**		
**Mode of fracture (%)**	**Control **	**Ultrasonic**	**File**	**Control **	**Ultrasonic**	**File**
**Adhesive fracture**	41.2	35.3	35.5	35.2	23.5	52.9
**Cohesive fracture**	11.8	23.5	11.8	17.6	23.5	5.9
**Mixed fracture**	47.1	41.1	52.9	47.1	52.9	41.2

These findings were consistent with the results of the studies of Guiotti *et al.* [7]. However other studies concluded that CH had no significant effect on the push out bond strength of sealers [12, 28-30]. This inconsistency may be due to different methodologies for CH removal. For instance, Üstün *et al.* [30] used 1% NaOCl irrigant with ultrasonic agitation. Amin *et al.* [28] used a ProTaper F5 file followed by passive ultrasonic irrigation with 2.5 % NaOCl with a final flush of 17 % EDTA for this purpose. 

When comparing the control specimens, the push out bond strength of AH-26 was higher than BC Sealer but the difference was not statistically significant. However, in specimens receiving CH, the push out bond strength of AH-26 was significantly higher than BC Sealer regardless of the CH removal technique. Al-Haddad *et al.* [31] confirmed the presence of higher percentage of gaps between BC Sealer and dentin. This may cause lower bond strength values in this sealer. Furthermore, it has been shown that the presence of moisture is essential for the setting of BC Sealer [10, 11]. Shokouhinejad *et al.* [32] attributed the lower bond strength of BC Sealer to the presence of inadequate moisture in root canals dried prior to obturation with this sealer. In our study the root canals were dried with paper points prior to obturation.

It should be noted that the clinical significance of this decrease in push out bond values and its effect on the outcome of endodontic treatments are not clear yet.

The dominant mode of failure in all experimental groups was mixed with an exception of BC Sealer specimens which undergone CH removal by hand files. In the latter, the dominant mode of failure was adhesive indicating that higher levels of residual CH in BC Sealer specimens affectively reduced the bond strength. Mixed mode of failure may also be due to uninform CH removal from root canal walls that must be determined with further research. Akcay *et al.* [29] and Gokturk *et al.* [33] reported the adhesive mode of failure to be dominant in specimens in both groups receiving or not receiving CH intra-canal medicament. Akcay *et al.* [29] used gutta-percha and AH-Plus Jet epoxy resin-based sealer with a single-cone technique of obturation. Presence of a thicker layer of sealer as seen in the case of single cone obturation can contribute to the failure towards adhesive mode [34]. Furthermore, both studies used NaOCl and EDTA for CH removal. These differences may be the reason for differences seen between their studies and the present this investigation.

## Conclusion

Residual CH on dentinal walls of the root canal negatively affects the push out bond strength of AH-26 and Endosequence BC Sealer.
